# Whole-Brain Map of Long-Range Monosynaptic Inputs to Different Cell Types in the Amygdala of the Mouse

**DOI:** 10.1007/s12264-020-00545-z

**Published:** 2020-07-20

**Authors:** Jia-Yu Fu, Xiao-Dan Yu, Yi Zhu, Shi-Ze Xie, Meng-Yu Tang, Bin Yu, Xiao-Ming Li

**Affiliations:** 1 Center for Neuroscience and Department of Neurology of Second Affiliated Hospital, Zhejiang University School of Medicine, Hangzhou, 310058 China; 2 NHC and CAMS Key Laboratory of Medical Neurobiology, Center for Brain Science and Brain-Inspired Intelligence, Guangdong-Hong Kong-Macao Greater Bay Area, Joint Institute for Genetics and Genome Medicine between Zhejiang University and University of Toronto, Hangzhou, 310058 China

**Keywords:** Basolateral amygdala, Central amygdala, Rabies virus retrograde tracing, Glutamatergic, GABAergic, Mouse

## Abstract

The amygdala, which is involved in various behaviors and emotions, is reported to connect with the whole brain. However, the long-range inputs of distinct cell types have not yet been defined. Here, we used a retrograde trans-synaptic rabies virus to generate a whole-brain map of inputs to the main cell types in the mouse amygdala. We identified 37 individual regions that projected to neurons expressing vesicular glutamate transporter 2, 78 regions to parvalbumin-expressing neurons, 104 regions to neurons expressing protein kinase C-δ, and 89 regions to somatostatin-expressing neurons. The amygdala received massive projections from the isocortex and striatum. Several nuclei, such as the caudate-putamen and the CA1 field of the hippocampus, exhibited input preferences to different cell types in the amygdala. Notably, we identified several novel input areas, including the substantia innominata and zona incerta. These findings provide anatomical evidence to help understand the precise connections and diverse functions of the amygdala.

**Electronic supplementary material:**

The online version of this article (10.1007/s12264-020-00545-z) contains supplementary material, which is available to authorized users.

## Introduction

As a hub responsible for the control of diverse behaviors and the modulation of different emotions such as fear, anxiety, and reward, the amygdala is reported to connect to a large number of brain regions [1–7]. The amygdala consists of the basolateral amygdala (BLA) and the central nucleus of the amygdala (CeA). The BLA receives projections from the medial geniculate nucleus, auditory cortex (Au), and medial prefrontal cortex (mPFC), and projects to various nuclei such as the nucleus accumbens, ventral hippocampus, prelimbic cortex, and CeA [8–13]. Previous studies have reported the long-range connectivity of the CeA, including inputs from the BLA, paraventricular thalamic nucleus (PVT), mPFC, bed nucleus of the stria terminalis (BST), and raphe nucleus, and output projections to the periaqueductal grey, nucleus tractus solitarius, locus coeruleus, and hypothalamus [14–16]. Although various studies have explored the innervation of the amygdala and its outputs, research on whole-brain inputs to specific cell types in the BLA and CeA is lacking.

**Table 1:** Abbreviations and classifications of brain structures.

Abbreviation	Name	Parent Brain Region
AAA	Anterior amygdalar area	Striatum
Acb	Accumbens nucleus, shell	Striatum
ACo	Anterior cortical amygdaloid nucleus	Olfactory areas
AD	Anterodorsal thalamic nucleus	Thalamus
ADP	Anterodorsal preoptic nucleus	Hypothalamus
AH	Anterior hypothalamic area	Hypothalamus
AHi	Amygdalohippocampal area	Hippocampal formation
AI	Agranular insular cortex	Isocortex
AM	Anteromedial thalamic nucleus	Thalamus
AOP	Anterior olfactory nucleus, posterior part	Olfactory areas
Apir	Amygdalopiriform transition area	Olfactory areas
APT	Anterior pretectal nucleus	Midbrain
Arc	Arcuate hypothalamic nucleus	Hypothalamus
Au	Auditory cortex	Isocortex
AV	Anteroventral thalamic nucleus	Thalamus
BIC	Nucleus of the brachium of the inferior colliculus	Fiber tracts
BST	Bed nucleus of the stria terminalis	Pallidum
CA1	Field CA1 of the hippocampus	Hippocampal formation
Cg	Cingulate cortex	Isocortex
CM	Central medial thalamic nucleus	Thalamus
Cpu	Caudate putamen (striatum)	Striatum
CxA	Cortex-amygdala transition zone	Olfactory areas
DG	Dentate gyrus	Hippocampal formation
DI	Dysgranular insular cortex	Isocortex
DLG	Dorsal lateral geniculate nucleus	Thalamus
DLO	Dorsolateral orbital cortex	Isocortex
DM	Dorsomedial hypothalamic nucleus	Hypothalamus
DP	Dorsal peduncular cortex	Olfactory areas
DpG	Deep gray layer of the superior colliculus	Midbrain
DpMe	Deep mesencephalic nucleus	Midbrain
DR	Dorsal raphe nucleus	Midbrain
DT	Dorsal terminal nucleus of the accessory optic tract	Midbrain
DTT	Dorsal tenia tecta	Fiber tracts
ECIC	External cortex of the inferior colliculus	Midbrain
Ect	Ectorhinal cortex	Isocortex
Ent	Entorhinal cortex	Hippocampal formation
EP	Endopiriform nucleus	Cortical subplate
Eth	Ethmoid thalamic nucleus	Thalamus
GI	Glomerular layer of the olfactory bulb	Olfactory areas
Gus	Gustatory thalamic nucleus	Thalamus
HDB	Nucleus of the horizontal limb of the diagonal band	Pallidum
HIP	Hippocampal region	Hippocampal formation
I	Intercalated nuclei of the amygdala	Striatum
IAD	Interanterodorsal thalamic nucleus	Thalamus
IAM	Interanteromedial thalamic nucleus	Thalamus
IF	Interfascicular nucleus	Midbrain
IGL	Intergeniculate leaf	Thalamus
IL	Infralimbic cortex	Isocortex
IM	Intercalated amygdaloid nucleus	Striatum
IMD	Intermediodorsal thalamic nucleus	Thalamus
InG	Intermediate gray layer of the superior colliculus	Midbrain
InWh	Intermediate white layer of the superior colliculus	Midbrain
IP	Interpeduncular nucleus	Midbrain
IPAC	Interstitial nucleus of the posterior limb of the anterior commissure	Striatum
LA	Lateroanterior hypothalamic nucleus	Hypothalamus
LEnt	Lateral entorhinal cortex	Hippocampal formation
LGP	Lateral globus pallidus	Pallidum
LH	Lateral hypothalamic area	Hypothalamus
LHb	Lateral habenular nucleus	Thalamus
LO	Lateral orbital cortex	Isocortex
LOT	Nucleus of the lateral olfactory tract	Olfactory areas
LP	Lateral posterior thalamic nucleus	Thalamus
LPB	Lateral parabrachial nucleus	Pons
LPO	Lateral preoptic area	Hypothalamus
LS	Lateral septal nucleus	Striatum
LSI	Lateral septal nucleus, intermediate part	Striatum
M1	Primary motor cortex	Isocortex
MCLH	Magnocellular nucleus of the lateral hypothalamus	Hypothalamus
MCPO	Magnocellular preoptic nucleus	Pallidum
MD	Mediodorsal thalamic nucleus	Thalamus
Me	Medial amygdaloid nucleus	Striatum
MG	Medial geniculate nucleus	Thalamus
MGP	Medial globus pallidus	Pallidum
MiTg	Microcellular tegmental nucleus	Midbrain
MnPO	Median preoptic nucleus	Hypothalamus
MnR	Median raphe nucleus	Midbrain
MO	Medial orbital cortex	Isocortex
MPA	Medial preoptic area	Hypothalamus
MPO	Medial preoptic nucleus	Hypothalamus
MS	Medial septal nucleus	Pallidum
Mtu	Medial tuberal nucleus	Hypothalamus
NDB	Diagonal band nucleus	Pallidum
OLF	Medial geniculate nucleus	Thalamus
op	Optic nerve layer of the superior colliculus	Midbrain
ORB	Orbital area	Isocortex
Pa	Paraventricular hypothalamic nucleus	Hypothalamus
Pa4	Paratrochlear nucleus	Midbrain
PAG	Periaqueductal gray	Midbrain
PAL	Pallidum	Pallidum
PB	Parabrachial nucleus	Pons
Pe	Periventricular hypothalamic nucleus	Hypothalamus
PERI	Perirhinal area	Isocortex
PF	Parafascicular thalamic nucleus	Thalamus
PH	Posterior hypothalamic area	Hypothalamus
PIL	Posterior intralaminar thalamic nucleus	Thalamus
Pir	Piriform cortex	Olfactory areas
PL	Paralemniscal nucleus	Fiber tracts
PLCo	Posterolateral cortical amygdaloid nucleus	Olfactory areas
PMCo	Posteromedial cortical amygdaloid nucleus	Olfactory areas
PN	Paranigral nucleus	Midbrain
Po	Posterior thalamic nuclear group	Thalamus
PP	Peripeduncular nucleus	Thalamus
PPT	Posterior pretectal nucleus	Midbrain
PPTg	Pedunculopontine tegmental nucleus	Midbrain
PR	Prerubral field	Hypothalamus
PRh	Perirhinal cortex	Isocortex
PrL	Prelimbic cortex	Isocortex
PSTh	Parasubthalamic nucleus	Hypothalamus
PT	Paratenial thalamic nucleus	Thalamus
pv	Periventricular fiber system	Hypothalamus
PVT	Paraventricular thalamic nucleus	Thalamus
Py	Pyramidal cell layer of the hippocampus	Hippocampal formation
RCh	Retrochiasmatic area	Hypothalamus
Re	Reuniens thalamic nucleus	Thalamus
Rh	Rhomboid thalamic nucleus	Thalamus
RLi	Rostral linear nucleus of the raphe	Midbrain
RR	Retrorubral nucleus	Midbrain
RRF	Retrorubral field	Midbrain
RSA	Retrosplenial agranular cortex	Isocortex
Rt	Reticular thalamic nucleus	Thalamus
S	Subiculum	Hippocampal formation
SS	Somatosensory cortex	Isocortex
SC	Superior colliculus	Midbrain
SCh	Suprachiasmatic nucleus	Hypothalamus
SFi	Septofimbrial nucleus	Striatum
SFO	Subfornical organ	Hypothalamus
SG	Suprageniculate thalamic nucleus	Thalamus
SI	Substantia innominata	Pallidum
SNC	Substantia nigra, compact part	Midbrain
SNL	Substantia nigra, lateral part	Midbrain
SNR	Substantia nigra, reticular part	Midbrain
SPF	Subparafascicular thalamic nucleus	Thalamus
STh	Subthalamic nucleus	Hypothalamus
Sub	Submedius thalamic nucleus	Thalamus
SubB	Subbrachial nucleus	Thalamus
SuG	Superficial gray layer of the superior colliculus	Midbrain
TC	Tuber cinereum area	Hypothalamus
TE	Terete hypothalamic nucleus	Hypothalamus
TeA	Temporal association cortex	Isocortex
TS	Triangular septal nucleus	Pallidum
Tu	Olfactory tubercle	Olfactory areas
VDB	Nucleus of the vertical limb of the diagonal band	Fiber tracts
VIS	Visual cortex	Isocortex
VLG	Ventral lateral geniculate nucleus	Thalamus
VLPO	Ventrolateral preoptic nucleus	Hypothalamus
VMH	Ventromedial hypothalamic nucleus	Hypothalamus
VMPO	Ventromedial preoptic nucleus	Hypothalamus
VO	Ventral orbital cortex	Isocortex
VPM	Ventral posteromedial thalamic nucleus	Thalamus
VTA	Ventral tegmental area	Midbrain
VTT	Ventral tenia tecta	Olfactory areas
ZI	Zona incerta	Hypothalamus

The BLA is composed of a majority (80%–85%) of spiny glutamatergic neurons and a minority (20%) of GABAergic neurons, including parvalbumin-expressing (PV^+^) interneurons [17–21]. These PV^+^ neurons constitute 19%–43% of GABAergic neurons in the BLA, and > 90% of PV^+^ neurons are GABAergic [22]. The CeA is a striatum-like structure consisting primarily of GABAergic neurons, which can be specifically distinguished by their molecular markers – those expressing protein kinase C-δ (PKC-δ^+^, ~ 50% of GABAergic neurons) and those expressing somatostatin (SST^+^, ~ 50% of GABAergic neurons – and these markers are rarely co-expressed [23–25]. For these reasons, we chose vesicular-glutamate transporter 2-positive (VGLUT2^+^) and PV^+^ neurons in the BLA and PKC-δ^+^ and SST^+^ neurons in the CeA as the main neuronal types in this study.

To provide anatomical evidence to help understand the precise connections and diverse functions of the amygdala, we developed a detailed map of the whole-brain long-range inputs to the amygdala and analyzed the distributions of inputs to the four main cell types in the amygdala.

## Methods

### Animals

All animal experiments were performed according to the guidelines of Zhejiang University for the care and use of laboratory animals. All protocols were approved by the Zhejiang University Animal Experimentation Committee. *VGLUT2-IRES-Cre* (no. 016963) [26], *PV-IRES-Cre* (no. 008069) [27], *SST-IRES-Cre* (no. 013044) [28], and *Ai14* mice (no. 007914) [29] were from the Jackson Laboratory (Bar Harbor, USA). The *PKC-δ-IRES-Cre* mice [30] were kindly provided by Prof. Hao-Hong Li (Huazhong University of Science and Technology, China). Adult male mice (4 per group) aged 2–4 months were used in the experiments. All mice were housed with food and water provided *ad libitum* under a 12-h dark-light cycle at 22 ± 1 °C and 55% ± 5% humidity.

### Viruses and Viral Injections

All viruses used in the trans-synaptic retrograde tracing experiments [rAAV-EF1α-DIO-RVG (5.53 × 10^12^ genomic copies/mL), rAAV-CAG-DIO-TVA-EGFP (5.22 × 10^12^ genomic copies/mL), and RV-ENVA-∆G-dsRed (3.0 × 10^8^ genomic copies/mL)] were provided by BrainVTA (Wuhan, China). For rabies virus tracing, rAAV-EF1α-DIO-RVG and rAAV-CAG-DIO-TVA-EGFP were mixed at a ratio of 1:1. A 100-nL mixture was unilaterally injected into the BLA or CeA of mice. Three weeks later, 200 nL of RV-ENVA-∆G-dsRed was injected in the same place. Mice were perfused one week later, and their brains were sectioned for fluorescence imaging.

### Animal Surgery

Mice were anesthetized with sodium pentobarbital (50 mg/kg, intraperitoneal injection). Surgery was performed with each mouse fixed in a stereotaxic frame (RWD Life Science, Shenzhen, China). The viral injection coordinates (in mm, from midline, bregma, and dorsal surface) were: BLA (2.95, − 0.96, − 4.95) and CeA (2.85, − 0.95, − 4.50).

### Histology and Imaging

Each mouse was anesthetized with sodium pentobarbital (100 mg/kg, intraperitoneal injection) and transcardially perfused with phosphate-buffered saline (PBS), followed by 4% paraformaldehyde in PBS. The brain was removed and post-fixed in 4% paraformaldehyde for 4–6 h at 4 °C, immersed in 30% sucrose (*w*/*v*) in PBS for 48 h, and then embedded in Optimal Cutting Temperature compound. Coronal cryosections were cut at 50 μm (Leica CM1950) and washed three times with PBS (5 min each).

For immunofluorescence staining, sections were blocked with 3% bovine serum albumin in PBST (0.3% Triton X-100 in PBS) for 1 h and incubated with primary antibodies overnight at 4 °C. After incubation, the sections were washed and incubated with a fluorescent dye-conjugated secondary antibody (1:400, Invitrogen, Carlsbad, USA) for 2 h at room temperature. The primary antibodies were anti-PV (1:1000, PV 27, Swant, Marly, Switzerland), anti-PKC-δ (1:500, #610398, BD Biosciences, San Jose, USA), and anti-SST (1:500, #20067, Immunostar, Hudson, USA). After staining with the nuclear dye 4,6-diamidino-2-phenylindole (Sigma-Aldrich, St. Louis, USA), the sections were used for imaging.

Confocal images were captured under a 20× objective (numerical aperture 1.2) using an A-1R confocal microscope (Nikon, Tokyo, Japan) and large images were captured under a 10× objective using a Virtual Slide microscope VS120 (Olympus, Tokyo, Japan).

### Cell Counts and Statistics

We sampled every fourth 50-μm section from bregma + 2.5 mm to − 5 mm. Each section was matched to the corresponding atlas level of the Allen Adult Mouse Brain Atlas and Allen Mouse Common Coordinate Framework and Reference Atlas [31, 32]. We then counted the number of dsRed-expressing neurons in individual nuclei within the entire brain of each mouse from rostral to caudal using ImageJ v1.52n software (NIH, Bethesda, USA) manually and blindly. Regions within ~ 600 μm of the injection site were omitted from the data (BLA, CeA, medial and basomedial amygdalar nuclei). All values are presented as the mean ± SEM. Two-way analysis of variance (ANOVA) was used for group differences and Sidak’s test was used for multiple comparisons with GraphPad Prism 6 v6.01 (GraphPad Software, San Diego, USA). Differences were considered statistically significant when *P* < 0.05.

## Results

### Experimental Strategy

**Fig. 1:**
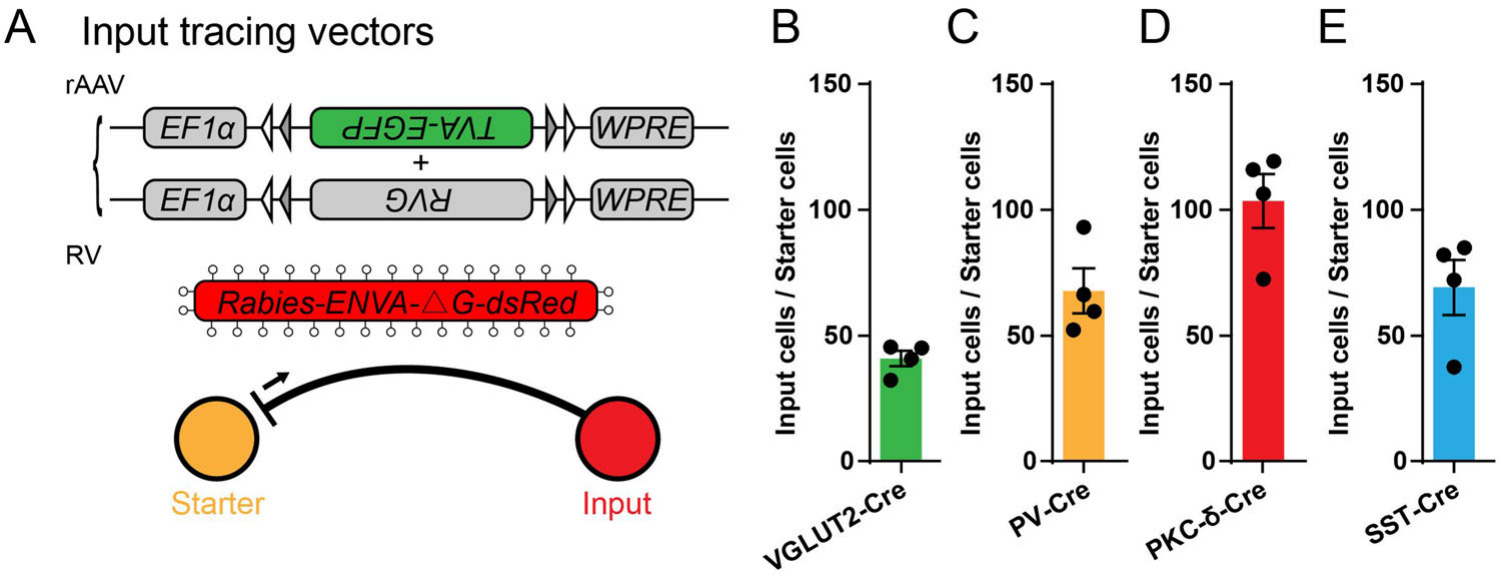
Virus injection strategy. **A** Schematic of viral vectors and rabies virus-mediated trans-synaptic retrograde tracing of amygdala inputs. **B**–**E** Ratios of input cells to starter cells in BLA VGLUT2-Cre mice (**B**), BLA PV-Cre mice (**C**), CeA PKC-δ-Cre mice (**D**), and CeA SST-Cre mice (**E**) (*n* *=* 4/group, mean ± SEM).

**Fig. 2:**
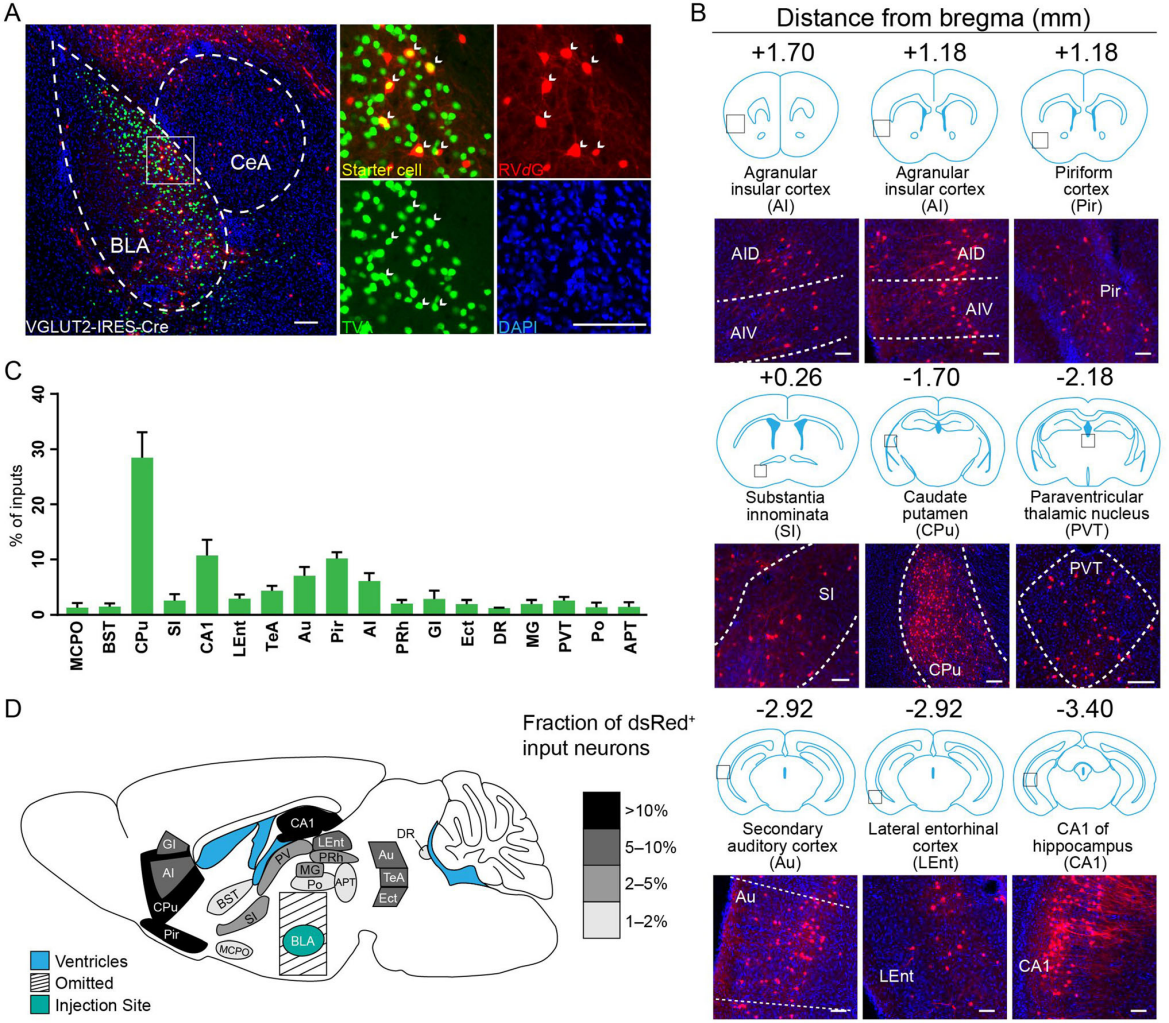
Long-range inputs to BLA VGLUT2^+^ neurons. **A** Representative images of starter cells restricted to the BLA. Starter cells expressed both GFP and dsRed fluorescent proteins were marked by arrowheads. Scale bars, 100 μm. **B** Representative images of RV-labeled input neurons to BLA VGLUT2^+^ neurons from selected nuclei. Scale bars, 100 μm. **C** Whole-brain distribution of input nuclei to BLA VGLUT2^+^ neurons with input percentages > 1% (mean ± SEM). **D** Schematic summary of brain regions providing the largest average fractional inputs to BLA VGLUT2^+^ neurons. Abbreviations are shown in Table 1.

**Fig. 3:**
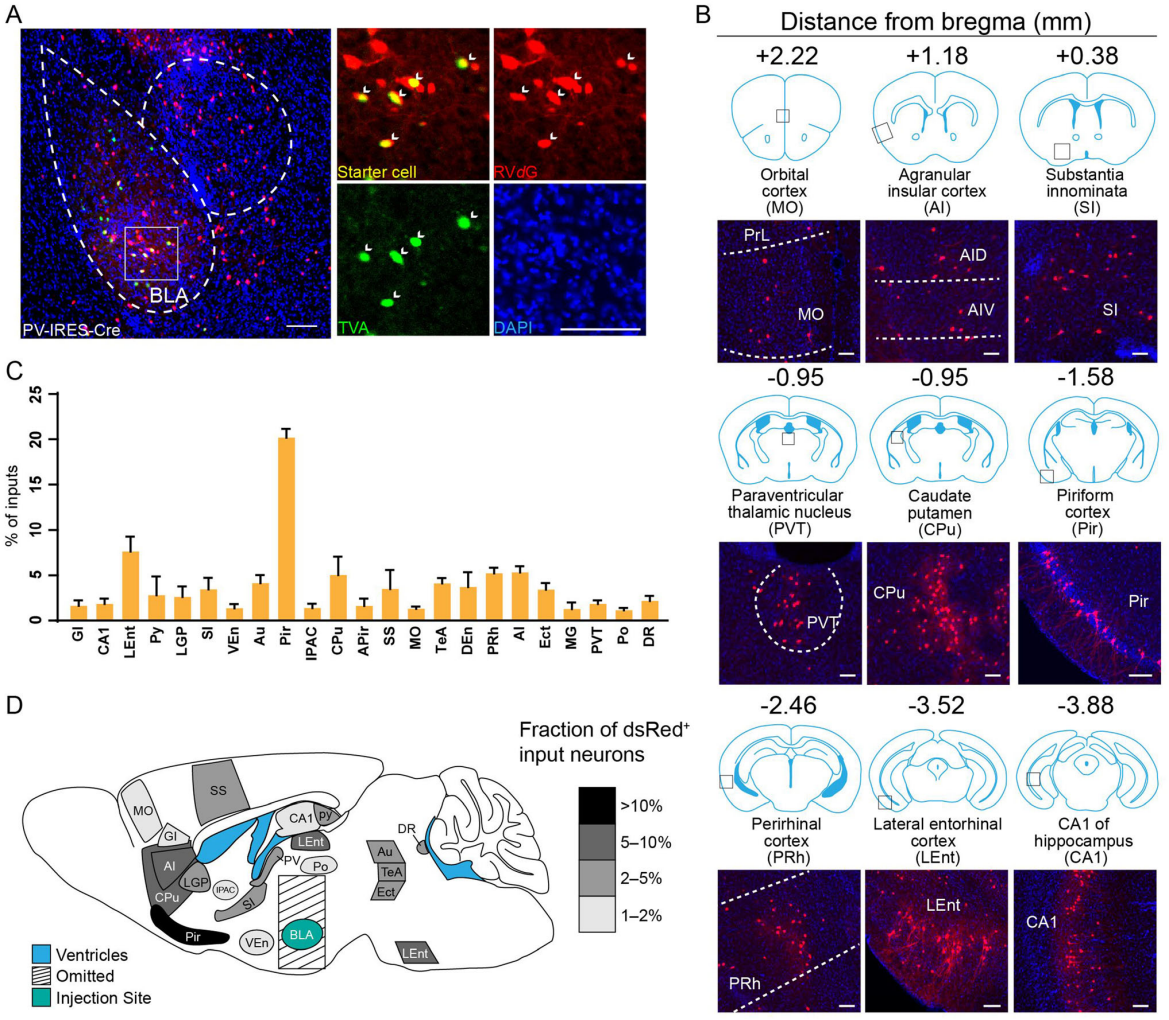
Long-range inputs to BLA PV^+^ GABAergic neurons. **A** Representative images of starter cells restricted to the BLA. Starter cells expressed both GFP and dsRed fluorescent proteins were marked by arrowheads. Scale bars, 100 μm. **B** Representative images of RV-labeled input neurons to BLA PV^+^ neurons from selected nuclei. Scale bars, 100 μm. **C** Whole-brain distribution of input nuclei to BLA PV^+^ neurons with input percentages > 1% (mean ± SEM). **D** Schematic summary of regions providing the largest average fractional inputs to BLA PV^+^ neurons. Abbreviations are shown in Table 1.

**Fig. 4:**
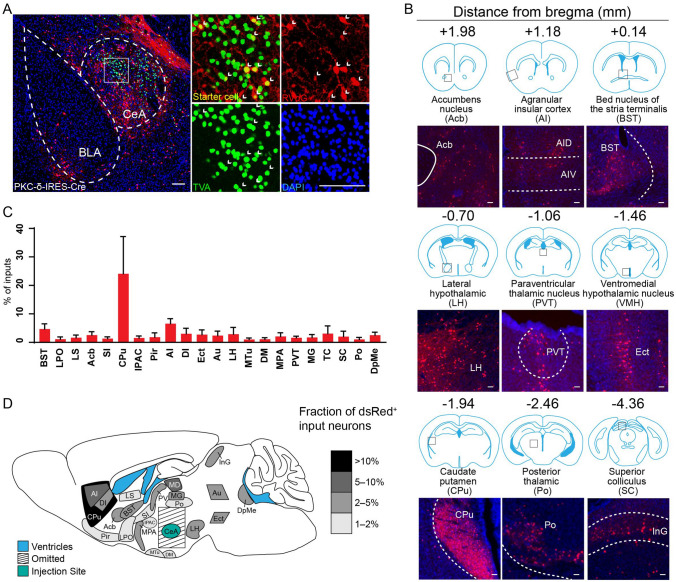
Long-range inputs to CeA PKC-δ^+^ GABAergic neurons. **A** Representative images of starter cells restricted to the CeA. Starter cells expressed both GFP and dsRed fluorescent proteins were marked by arrowheads. Scale bars, 100 μm. **B** Representative images of RV-labeled input neurons to CeA PKC-δ^+^ neurons from selected nuclei. Scale bars, 100 μm. **C** Whole-brain distribution of input nuclei to CeA PKC-δ^+^ neurons with input percentages > 1% (mean ± SEM). **D** Schematic summary of regions providing the largest average fractional inputs to CeA PKC-δ^+^ neurons. Abbreviations are shown in Table 1.

**Fig. 5:**
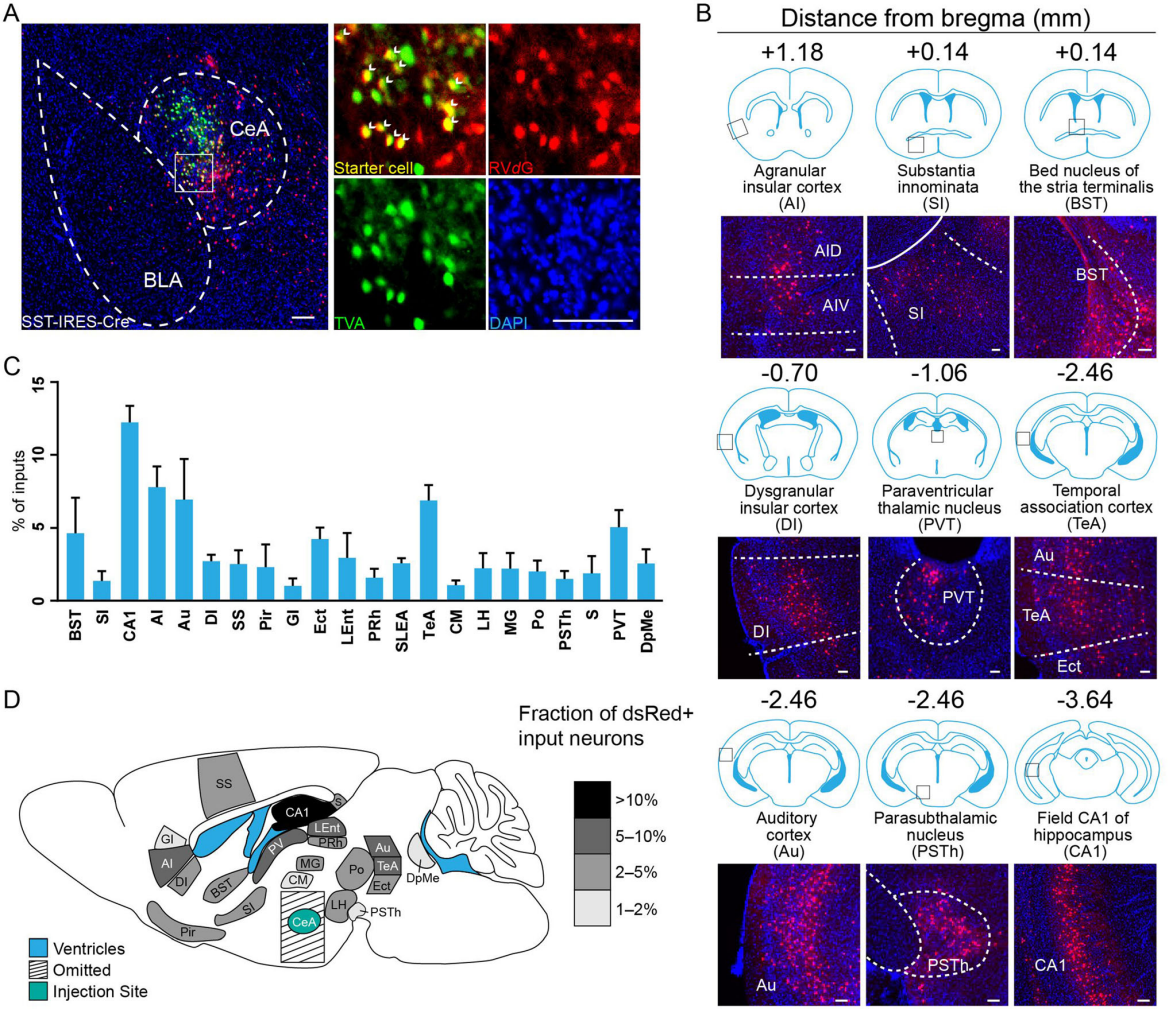
Long-range inputs to CeA SST^+^ GABAergic neurons. **A** Representative images of starter cells restricted to the CeA. Starter cells expressed both GFP and dsRed fluorescent proteins were marked by arrowheads. Scale bars, 100 μm. **B** Representative images of RV-labeled input neurons to CeA SST^+^ neurons from selected nuclei. Scale bars, 100 μm. **C** Whole-brain distribution of input nuclei to CeA SST^+^ neurons with input percentages > 1% (mean ± SEM). **D** Schematic summary of regions providing the largest average fractional inputs to CeA SST^+^ neurons. Abbreviations are shown in Table 1.

To build a cell-type-specific whole-brain map of inputs to the BLA and CeA, we used four mouse strains: *Vesicular-Glutamate-Transporter-2-IRES-Cre* mice (*VGLUT2-IRES-Cre*) for VGLUT2^+^ glutamatergic neurons in the BLA, *Parvalbumin-IRES-Cre* mice (*PV-IRES-Cre*) for PV^+^ neurons in the BLA, and *Protein-Kinase-C-δ-IRES-Cre* mice (*PKC-δ-IRES-Cre*) and *Somatostatin-IRES-Cre* mice (*SST-IRES-Cre*) for the two major GABAergic subtypes in the CeA. First, specific Cre-positive neurons were infected with two Cre-dependent rAAVs that expressed TVA receptor-EGFP and rabies glycoprotein (RVG). Second, ENVA-pseudotyped RVG-deleted rabies virus (dsRed) specifically infected TVA-expressing cells, and only spread when RVG was provided [33]. The experimental strategies are shown in Fig. 1A. Neurons expressing both dsRed and EGFP were labeled as starter cells. Representative images for the starter cells of each Cre line mouse are shown in Figs 2A, 3A, 4A, and 5A. One week after the rabies virus injection, the mice were sacrificed and whole-brain sections were cut for imaging. For each cell type, we injected 5–6 mice with the rabies virus. Only those mice in which starter cells were specifically expressed in the target region were counted (Fig. S1).

In control experiments, we injected the same two helper viruses into the BLA or CeA of wild-type mice (*n* *=* 3/group), followed by the same rabies viruses injected into the same place three weeks later to test the leakage of the rabies virus-tracing system. There was no labeling of either EGFP or dsRed in the BLA or CeA (Fig. S2). These data indicated that the rabies virus did not infect Cre-negative neurons in our experiments. To confirm the selectivity of Cre recombinase, we crossed Cre mice with Ai14 mice, followed by staining with cell-type-specific markers (Fig. S3). We found that only a few marker-negative neurons (4.7%–7.0%) expressed Cre recombinase in all three strains.

We calculated the ratio between the number of input cells and starter cells. The results showed that different cell subtypes in the BLA and CeA received a comparable number of input cells per starter cell (Fig. 1B–E). The numbers of starter and input cells are shown in Supplementary Table S3. We provide input nuclei with input percentages > 1% (defined as percentage of input cells in each nucleus to total input cells throughout the whole brain) for BLA VGLUT2^+^ mice, BLA PV^+^ mice, CeA PKC-δ^+^ mice, and CeA SST^+^ mice in Figs 2, 3, 4, 5. Many upstream nuclei with input percentages  < 1% were traced and are listed in Supplementary Table S1.

### Identification of Major Long-range Inputs to BLA VGLUT2^+^ Glutamatergic Neurons

**Fig. 6:**
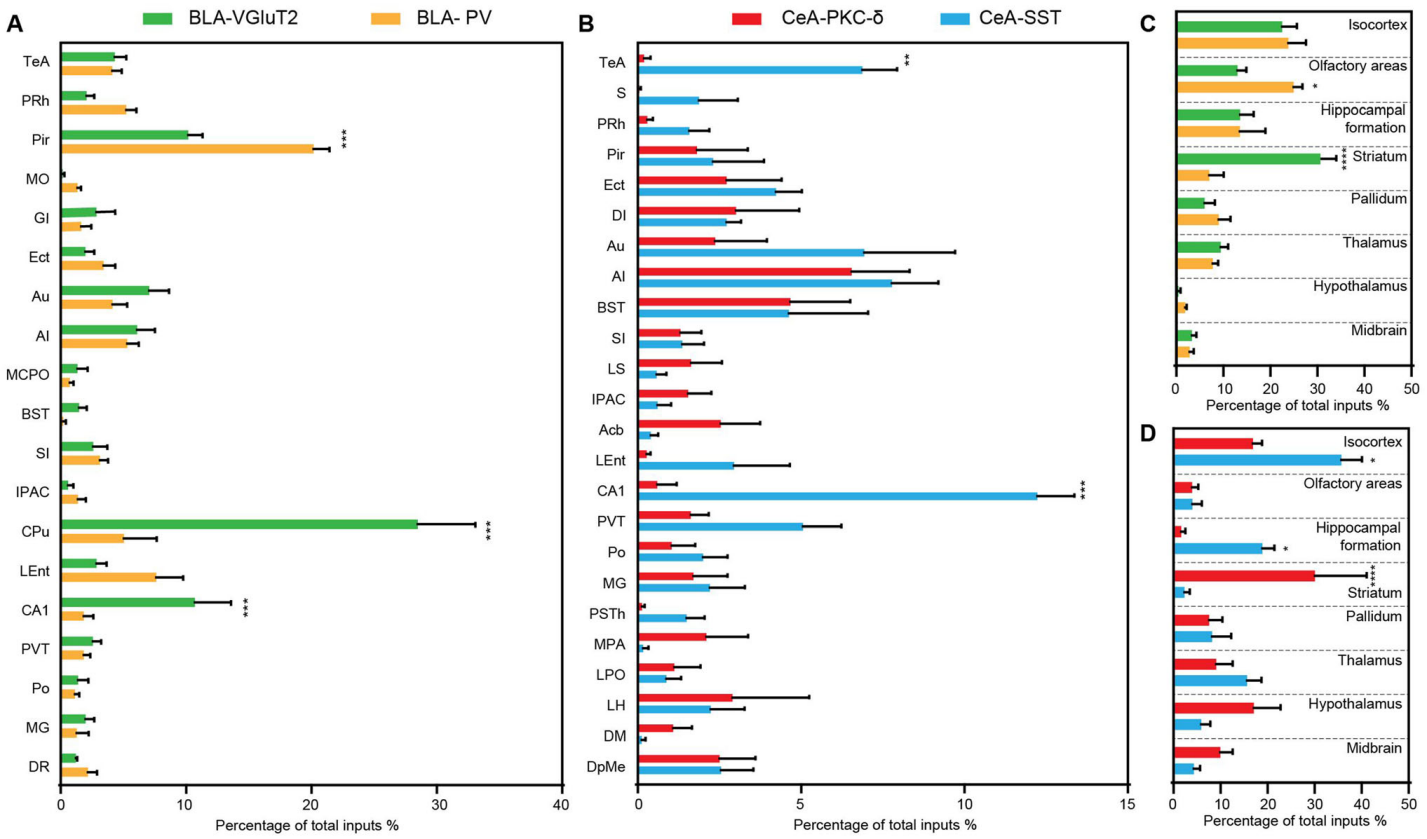
Comparison of input percentages of different nuclei among different cell types. **A** Comparison of input percentages from different nuclei in *VGLUT2-IRES-Cre* and *PV-IRES-Cre* mice (*n* *=* 4, ***P < 0.001, mean ± SEM). **B** Comparison of input percentages in *PKC-δ-IRES-Cre* and *SST-IRES-Cre* mice (*n* *=* 4, **P < 0.01, ***P < 0.001). **C** Percentage of total inputs from selected regions for BLA glutamatergic and PV^+^ neurons (*n* *=* 4, *P < 0.05, ****P < 0.0001). **D** Percentage of total inputs from selected regions for CeA PKC-δ^+^ and SST^+^ neurons (*n* *=* 4, *P < 0.05, ****P < 0.0001). Abbreviations are shown in Table 1.

Whole-brain mapping of RV-dsRed-labeled neurons revealed inputs from 37 discrete regions to BLA glutamatergic neurons. Those input nuclei with percentages > 1% are listed in Fig. 2C. Dense long-range inputs with dsRed-labeled cells were observed in the caudate-putamen (CPu), the CA1 field of the hippocampus (CA1), piriform cortex (Pir), Au, agranular insular cortex (AI), lateral entorhinal cortex, PVT, and substantia innominata (SI) (Fig. 2B). We dissected all the input nuclei into eight areas. The majority of input nuclei were located in the striatum (30.67% ± 3.38%), followed by the isocortex (22.52% ± 3.16%), hippocampal formation (13.57% ± 2.88%), olfactory areas (13.03% ± 1.87%), thalamus (9.49% ± 1.54%), pallidum (6.05% ± 2.16%), midbrain (3.35% ± 0.95%), and hypothalamus (1.24% ± 0.74%) (Fig. 6C).

### Identification of Major Long-range Inputs to BLA PV^+^ GABAergic Interneurons

Whole-brain mapping of RV-dsRed-labeled neurons revealed inputs from 78 discrete regions to BLA PV^+^ neurons. Those input nuclei with percentages > 1% are listed in Fig. 3C. Dense long-range inputs with dsRed-labeled cells were observed in the Pir, lateral entorhinal cortex, perirhinal cortex, AI, medial orbital cortex, PVT, CPu, SI, and CA1 (Fig. 3B). We dissected all input nuclei into eight areas. The majority of input nuclei were located in olfactory areas (24.98% ± 1.88%), followed by the isocortex (23.85% ± 3.73%), hippocampal formation (13.51%± 5.42%), pallidum (9.01% ± 2.52%), thalamus (7.80% ± 1.09%), striatum (7.04% ± 3.04%), midbrain (2.82% ± 0.91%), and hypothalamus (1.88% ± 0.34%) (Fig. 6C).

### Identification of Major Long-range Inputs to CeA PKC-δ^+^ GABAergic Neurons

Whole-brain mapping of RV-dsRed-labeled neurons revealed inputs from 104 discrete regions to CeA PKC-δ^+^ neurons. Those input nuclei with percentages > 1% are listed in Fig. 4C. Dense long-range inputs with dsRed-labeled cells were observed in the CPu, AI, BST, PVT, ventromedial hypothalamic nucleus, posterior thalamic nuclear group, superior colliculus, and nucleus accumbens (Fig. 4B). We dissected all input nuclei into eight areas. The majority of input nuclei were located in the striatum (30.11% ± 10.96%), followed by the hypothalamus (17.07% ± 5.71%), isocortex (16.90% ± 1.92%), midbrain (9.94 ± 2.66%), thalamus (9.10% ± 3.49%), pallidum (7.60% ± 2.80%), olfactory areas (3.99% ± 1.32%), and hippocampal formation (1.64% ± 0.88%) (Fig. 6D).

### Identification of Major Long-range Inputs to CeA SST^+^ GABAergic Neurons

Whole-brain mapping of RV-dsRed-labeled neurons revealed inputs from 89 discrete regions to CeA SST^+^ neurons. Those input nuclei with percentages > 1% are listed in Fig. 5C. Dense long-range inputs with dsRed-labeled cells from the anterior to posterior brain were observed in CA1, AI, Au, temporal association cortex, PVT, BST, SI, parasubthalamic nucleus, and dysgranular insular cortex (Fig. 5B). We dissected all input nuclei into eight areas. The majority of input nuclei were located in the isocortex (35.72% ± 4.34%), followed by the hippocampal formation (18.92% ± 2.52%), thalamus (15.65% ± 3.05%), pallidum (8.25% ± 4.01%), hypothalamus (5.93% ± 1.91%), midbrain (4.36% ± 1.31%), olfactory areas (4.02% ± 2.01%), and striatum (2.32% ± 1.13%) (Fig. 6D).

### Comparison of Inputs between Different Cell Types

To determine the quantitative differences in input distributions among different cells and subzones, we compared the input percentages of each nucleus between VGLUT2^+^ and PV^+^ neurons in the BLA (Fig. 6A) and between PKC-δ^+^ and SST^+^ GABAergic neurons in the CeA (Fig. 6B). Taking all regions (percentages > 1%) into calculation, the VLGUT2^+^ neurons received more projections from the CPu and CA1 than the PV^+^ neurons (*P* < 0.0001, *P* < 0.0001, Fig. 6A), whereas the Pir sent more projections to the PV^+^ neurons than to the VGLUT2^+^ neurons in the BLA (*P* < 0.0001, Fig. 6A). We also found that the SST^+^ neurons received more projections from the temporal association cortex and CA1 than the PKC-δ^+^ neurons (*P* *=* 0.002, *P* < 0.0001, Fig. 6B). Although the distribution was highly variable, the input percentages of some brain regions, such as the AI, Au, and SI, showed no significant differences.

We divided the whole brain into eight areas according to the Allen Mouse Common Coordinate Framework and Reference Atlas and calculated the input percentages among the different brain areas (Fig. 6C, D). Most input projections to the amygdala came from the cortex, and significant differences were found among the four cell types. In detail, there were significantly higher input percentages from the olfactory areas to the BLA PV^+^ neurons than to the BLA VGLUT2^+^ interneurons (*P* *=* 0.0155), and the striatum sent significantly more projections to the BLA VGLUT2^+^ interneurons than to the BLA PV^+^ neurons (*P* < 0.0001, Fig. 6C). The CeA SST^+^ neurons received more inputs than the CeA PKC-δ^+^ neurons from the isocortex (*P* *=* 0.0114) and hippocampal formation (*P* *=* 0.0250, Fig. 6D). In addition, the striatum sent more projections to the CeA PKC-δ^+^ neurons than to the SST^+^ neurons (*P* < 0.0001, Fig. 6D).

We found several novel projections not reported previously. The BLA VGLUT2^+^ and PV^+^ neurons both received projections from the SI (2.60% and 4.58%, respectively). The CeA PKC-δ^+^ neurons received projections from the SI (1.93%), deep mesencephalic nucleus (2.51%), tuber cinereum area (3.09%), and zona incerta (ZI) (0.80%). The CeA SST^+^ neurons received projections from the SI (1.97%) and deep mesencephalic nucleus (2.55%).

## Discussion

We used a modified rabies virus retrograde tracing system to map whole-brain long-range inputs to the BLA and CeA. In total, 37 individual brain regions projected to the BLA VGLUT2^+^ glutamatergic neurons and 78 regions projected to the BLA PV^+^ GABAergic neurons. In the CeA, PKC-δ^+^ GABAergic neurons received projections from 104 regions and SST^+^ GABAergic neurons received innervation from 89 regions. These data together built a detailed map of the long-range projections from the whole brain to specific neuronal types in the amygdala.

Our trans-synaptic tracing data demonstrated that several nuclei exhibited input preferences to different cell types in the amygdala. For example, we found that the BLA VGLUT2^+^ neurons received significantly more projections from CA1 than the PV^+^ neurons. Considering the role that projections from CA1 to the BLA play in fear conditioning and the massive intrinsic connections in the amygdala, this projection preference possibly indicates that glutamatergic neurons are the main input sites for sensory information, and PV^+^ interneurons are the modulators of principal neurons in the BLA [17, 34–36]. In addition, our data showed that the PV^+^ neurons received more projections from the Pir than the VGLUT2^+^ neurons. The Pir has a unique bidirectional connection with the BLA, which may be important in the association of different meanings with different odors [37]. Our findings suggest that PV^+^ neurons may be involved in odor information processing [36, 38]. In the CeA, we found that SST^+^ neurons received more projections from CA1 than PKC-δ^+^ neurons. Projections from CA1 to the CeA are necessary for the context-dependent retrieval of cued fear memories [35]. Considering the different roles of PKC-δ^+^ and SST^+^ neurons in conditioned fear [17, 39], these data suggest that CeA SST^+^ neurons are strongly associated with the retrieval of cued fear memories related to the hippocampus. Thus, these projection preferences indicate differences in connections and functions between different types of amygdala neurons.

The BLA is widely considered to be the sensory gateway to the amygdala [40]. As expected, our tracing results showed projections from the mPFC – consisting of the prelimbic and infralimbic cortex – to BLA PV^+^ interneurons (Table S1), in accordance with a prior study showing that opto-activation of the mPFC-to-BLA projection increases food intake behavior in mice [41]. The CeA is an important region of the amygdala, orchestrating a diverse set of behaviors, including fear, anxiety, and defensive responses [2, 42, 43]. Our rabies virus input-tracing identified several principal input regions reported in previous studies, such as the PVT [44], AI [45], BST [46], and substantia nigra pars compacta [47] (Figs 4 and 5, Table S1). Projections from the lateral parabrachial nucleus to the CeA may be essential for sodium intake [48–50]. We observed that the lateral parabrachial neurons were labeled with RV-dsRed in the SST^+^ neuron retrograde tracing (Table S1), indicating that SST^+^ neurons may be involved in salt appetitive behavior. Our results help to understand the heterogeneity of the amygdala and provide potential directions for further studies.

With the application of the rabies virus retrograde-tracing system, we identified several novel projections to the amygdala that have not been reported previously, including projections from the SI to both CeA PKC-δ^+^ and SST^+^ neurons (Figs 4 and 5). The lateral central amygdala (CeL) PKC-δ^+^-to-SI neural circuit modulates negative reinforcement learning [51]. Our findings suggest that the SI may have bidirectional connections with the CeA GABAergic neurons, possibly extending the roles of the latter in encoding motivation, fear conditioning, and conditioned reinforcement [51]. Interestingly, our trans-synaptic tracing results demonstrated a ZI-to-CeA PKC-δ^+^ neural circuit (Table S1). Stimulation of the ZI can be used to treat fear generalization [52]. Combined with recent findings on the functions of the ZI (eating, hunting, and sleeping) [53] and the role of CeA PKC-δ^+^ neurons in conditioned fear [17, 30], these novel projections may provide another explanation for how the ZI reacts in fear modulation and extend the potential roles of the amygdala in different functions. However, further optogenetic and electrophysiological studies are required to clarify these questions.

Previous study has revealed that direct glutamatergic projections from the cingulate cortex to the BLA participate in innate fear [54]. Our study showed that the BLA VGLUT2^+^ neurons did not receive inputs from the cingulate, whereas the PV^+^ neurons did. This discrepancy could be attributed to the different subtypes studied in the BLA. We did not cover other glutamatergic neuronal subtypes, such as VGLUT1^+^ glutamatergic neurons. The PV^+^ interneurons in the BLA can be divided into four subtypes based on their firing properties, and exhibit subtype-specific heterogeneity in their patterns of local synaptic connections, as well as in CeL early-spiking and late-spiking neurons [55]. It is difficult to distinguish the heterogeneities of these PV^+^ CeL early-spiking and late-spiking neurons in long-range connections with rabies virus tracing due to a lack of specific molecular markers for these subtypes. Thus, further studies combined with optogenetics and electrophysiological analysis are required to solve this issue. Using immunohistochemical and cholera toxin B tracing methods, previous studies have shown that the central and basal amygdala are targets of dopaminergic terminals [56–60]. We observed inputs from the mesencephalic dopaminergic system, such as from the substantia nigra and ventral tegmental area (VTA) to the BLA PV^+^ neurons (< 1%, see Table S1) and from the substantia nigra pars compacta, VTA, periaqueductal gray, and dorsal raphe to the CeA PKC-δ^+^ and SST^+^ neurons (< 1%, see Table S1), supporting the idea that these dopaminergic areas may form synapses in the amygdala. However, these sparse inputs could be the result of the limitations of the monosynaptic rabies virus-tracing system used in neuromodulatory projections such as dopaminergic transmission. It is likely that the extracellular space between the dopaminergic terminals and neurons in the amygdala does not allow the rabies virus particles to traverse effectively [61]. Further studies are required to test this possibility.

There are some limitations in this study. First, it was difficult to obtain the complete olfactory bulb when removing the brain, so we did not count input cells in the olfactory bulb. Second, the amygdala has very strong intranuclear and internuclear connectivity, such as separate projections from the BLA to CeA PKC-δ^+^ and SST^+^ neurons [17]. However, due to the leakage of rabies virus at the injection site, the intra-amygdala connections were excluded from our data, and regions around the BLA and CeA were omitted from analysis (e.g., the medial and basomedial amygdalar nuclei) [62]. Third, due to the limitation of transgenic mouse strains, we could not cover all neuronal subtypes in the amygdala, e.g., calbindin and calretinin GABAergic neurons in the BLA [22] and corticotropin-releasing factor and neurotensin-expressing neurons in the CeA [25]. As such, additional research on these important neuronal subtypes is required to address this issue.

Taking the above data into consideration, most afferent inputs to the amygdala came from the cortex, striatum, hippocampus, and thalamus, with great variation among the different cell types. Our research supports the idea of the amygdala as a node that contributes to multiple behaviors, as it received a varied and wide range of input projections from the whole brain. Furthermore, our study revealed a wide range of inputs to different molecular marker-labeled neurons in the amygdala. These findings will help to fully understand the roles of glutamatergic neurons and different GABAergic subtypes in the amygdala in different behaviors.

## Electronic supplementary material

Below is the link to the electronic supplementary material.

Supplementary material 1 (PDF 701 kb)
