# Supplementary material for: Whole-Brain Map of Long-Range Monosynaptic Inputs to Different Cell Types in the Amygdala of the Mouse
Source: Neurosci Bull. 2020 Jul 20;36(11):1381–94. doi: 10.1007/s12264-020-00545-z (PMC7674542; doi:10.1007/s12264-020-00545-z)
Supplement: Supplementary file 1 — Supplementary material 1 (PDF 701 kb) [file 12264_2020_545_MOESM1_ESM.pdf]

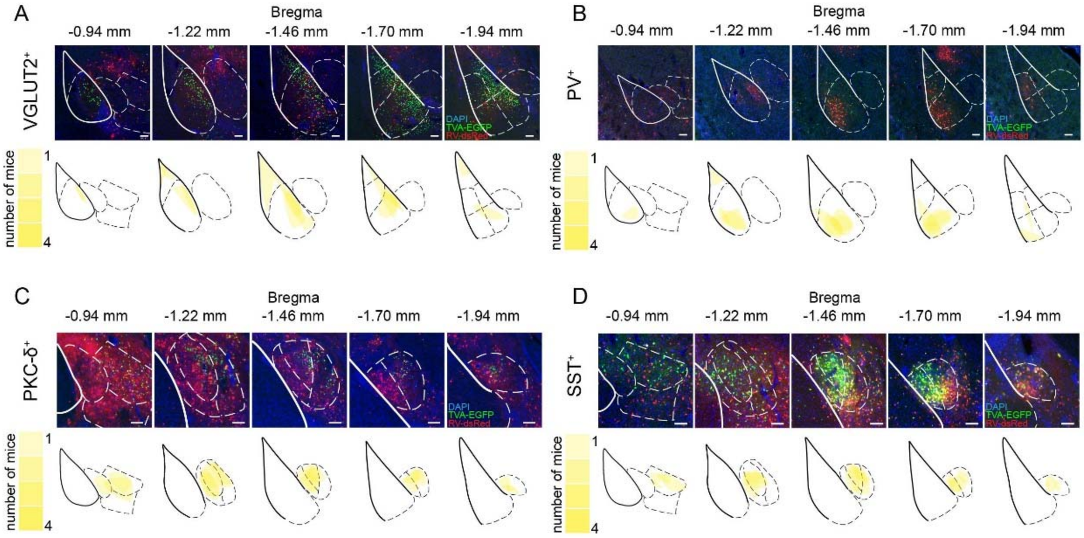

Fig. S1 Starter cell injection sites. **A**–**D** Representative images (upper) and overlays (lower) of starter cells from rostral to caudal BLA in one *VGLUT2-IRES-Cre* mouse (**A**), from rostral to caudal BLA in one *PV-IRES-Cre* mouse (**B**), from rostral to caudal CeA in one *PKC-δ-IRES-Cre* mouse (**C**), and from rostral to caudal CeA in one *SST-IRES-Cre* mouse (n = 4 mice/group; scale bars, 100 μm).

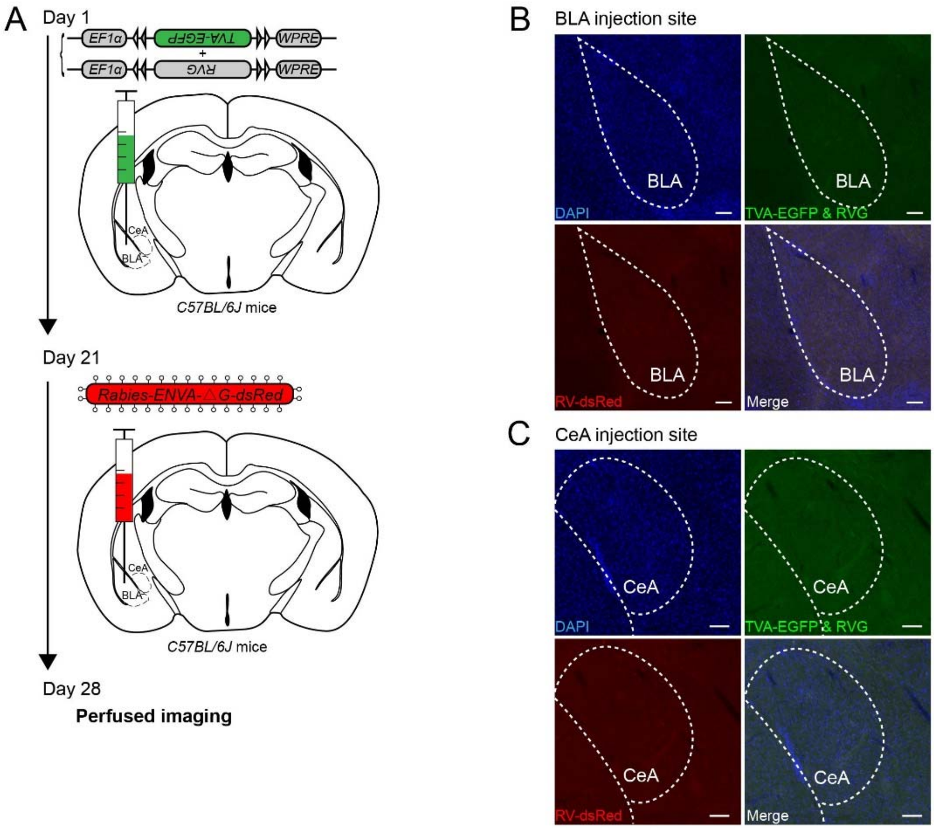

D

	Number of mice	Number of labeled cells in BLA / CeA
Wild type BLA injection	3	0
Wild type CeA injection	3	0

Fig. S2 Control experiment demonstrating the specificity of the retrograde trans-synaptic tracing approach. **A** Timeline of virus injection into the BLA or CeA of wild-type mice for retrograde trans-synaptic tracing. **B** Representative images of BLA neurons in wild-type mice. No TVA-EGFP or RV-dsRed fluorescence-positive cells were observed. Scale bars, 100 μm. **C** Representative images of CeA neurons in wild-type mice. No TVA-EGFP or RV-dsRed fluorescence-positive cells were observed. Scale bars, 100 μm. **D** Quantification of labeled cells in BLA and CeA.

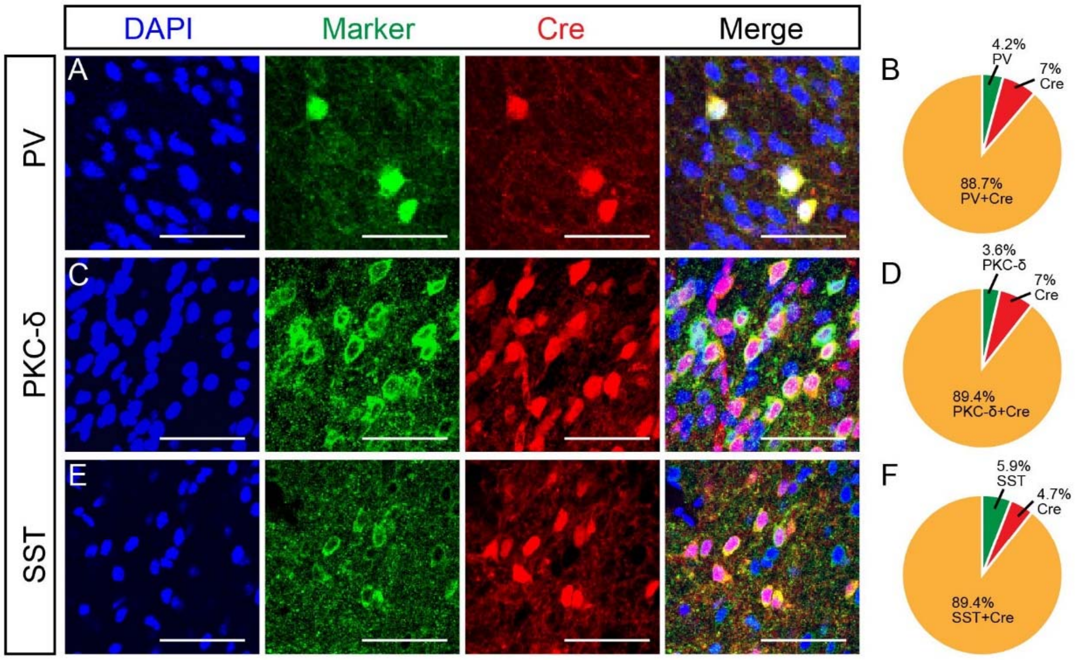

G

Mouse number	Co-expressed	Marker only	Cre only
PV::Ai14 #1	72	6	8
PV::Ai14 #2	116	2	8
PV::Ai14 #3	64	4	4
PKC-δ::Ai14 #1	490	16	30
PKC-δ::Ai14 #2	212	12	18
PKC-δ::Ai14 #3	244	10	26
SST::Ai14 #1	250	22	16
SST::Ai14 #2	284	14	14
SST::Ai14 #3	224	14	10

Fig. S3 Co-expression of Cre recombinase and cell-type-specific markers. **A** Representative images of BLA PV^+ neurons co-expressing Cre recombinase from *PV-Cre::Ai14* mice. Scale bars, 50 μm. **B** Pie chart representing fractions of PV-positive, Cre-positive, and co-expressing neurons in BLA (*n* = 3 mice). **C** Representative images of CeA PKC-δ^+ neurons co-expressing Cre recombinase from *PKC-δ-Cre::Ai14* mice. Scale bars, 50 μm. **D** Pie chart representing fractions of PKC-δ-positive, Cre-positive, and co-expressing neurons in CeA (*n* = 3 mice). **E** Representative images of CeA SST^+ neurons co-expressing Cre

recombinase from *SST-Cre::Ai14* mice. Scale bars, 50 μm. **F** Pie chart representing fractions of SST-positive, Cre-positive, and co-expressing neurons in CeA (n = 3 mice). **G** Numbers of neurons in each Cre::Ai14 mouse.

Table S1. List of brain areas (<1% of total inputs).

					VGLUT2$^+$					
	AAA	Acb	CM	DpMe	DTT	HDB	IL	IMD	IPAC	LH
Mean (%)	0.8348	0.7479	0.9457	0.4454	0.0731	0.649	0.05406	0.5858	0.5981	0.3028
	LPO	MD	MO	PAG	PF	PrL	SPF	VIS	VPM	
Mean (%)	0.1566	0.7551	0.1462	0.2611	0.2515	0.2071	0.6161	0.5092	0.9774	
					PV$^+$					
	AAA	AD	Ahi	AH	AM	AOP	BST	C1	Cg	CM
Mean (%)	0.5648	0.05479	0.3488	0.06849	0.04795	0.0411	0.2055	0.1575	0.09091	0.5908
	CxA	DI	DP	DTT	Eth	HDB	I	IL	IMD	IP
Mean (%)	0.5671	0.1438	0.04795	0.0137	0.03425	0.4384	0.06164	0.02055	0.441	0.08991
	LH	LO	LOT	LPO	MCPO	MD	MG	MnPO	MO	MPA
Mean (%)	0.7136	0.006849	0.09589	0.22	0.7361	0.289	0.4033	0.0274	0.03425	0.119
	MS	Pa4	PAG	PF	PIL	PMCo	PPTg	PrL	PSTh	PT
Mean (%)	0.0274	0.006849	0.0137	0.7889	0.2192	0.5137	0.04795	0.0411	0.2955	0.8207
	Re	Rh	S	SG	SNC	SNR	STh	Tu	VIS	VDB
Mean (%)	0.05479	0.0137	0.894	0.05479	0.3452	0.197	0.3322	0.2731	0.1334	0.06849
	VMPO	VPM	VTA	VTT	ZI					
Mean (%)	0.09849	0.116	0.03425	0.0137	0.07721					
					PKC-δ$^+$					
	AAA	ACo	ADP	AH	Ahi	Apir	APT	Arc	BIC	CA1
Mean (%)	0.2834	0.01134	0.09296	0.1849	0.5759	0.883	0.1601	0.9455	0.1439	0.5979
	Cg	DLG	DLO	DP	DpG	DR	DTT	ECIC	HDB	IF
Mean (%)	0.08163	0.4451	0.01587	0.5416	0.1905	0.5332	0.1671	0.1956	0.2426	0.05442
	IGL	IL	InWh	IP	LA	Lent	LGP	LHb	LO	M1
Mean (%)	0.4195	0.4273	0.1043	0.3537	0.2041	0.2726	0.3174	0.8321	0.295	0.04761
	MCPO	Me	MnPO	MnR	MO	MS	op	Pa	PAG	Pe
Mean (%)	0.2539	0.3061	0.1859	0.1002	0.2903	0.1791	0.1519	0.3673	0.9515	0.3605
	PF	PH	PIL	PLCo	PMCo	PN	PP	PPT	PR	PRh
Mean (%)	0.4815	0.8583	0.2653	0.09296	0.653	0.3378	0.3117	0.04081	0.07256	0.2933
	PrL	PSTh	PT	pv	py	RCH	RIi	RRF	RSA	Rt
Mean (%)	0.205	0.1337	0.2086	0.6371	0.1451	0.1519	0.04599	0.6703	0.2222	0.2744
	S	S1	SC	SCh	SFI	SFO	SG	SNC	SNL	SPF
Mean (%)	0.04535	0.7587	0.5893	0.297	0.03628	0.03401	0.1315	0.5966	0.02721	0.02721
	SubB	SuG	TeA	VIS	VDB	VLG	VLPO	VMH	VO	VPM
Mean (%)	0.1886	0.06122	0.1973	0.8998	0.03401	0.8221	0.288	0.7651	0.1756	0.3157
	VTA	ZI								
Mean (%)	0.5984	0.7958								
					SST$^+$					
	AAA	Acb	AD	AH	Apir	AV	BIC	Cg	DG	DM
Mean (%)	0.26	0.4022	0.07161	0.2628	0.237	0.09717	0.3091	0.06562	0.1389	0.1183
	DP	DR	DT	EP	Eth	Gus	HDB	IAD	IAM	IL
Mean (%)	0.4517	0.6637	0.137	0.8378	0.07407	0.2579	0.7936	0.0537	0.1683	0.1991
	IM	IMD	IPAC	LA	LGP	LO	LP	LPO	LS	M1
Mean (%)	0.1602	0.2647	0.609	0.2521	0.102	0.05778	0.6959	0.8796	0.5772	0.6312
	MCLH	MCPO	MD	Me	MiTg	MO	MPA	MPO	MS	NDB
Mean (%)	0.04814	0.3029	0.5977	0.3099	0.01576	0.6176	0.1623	0.1287	0.1662	0.1051
	ORB	PAG	PAL	PB	PERI	PF	PH	PIL	PL	PP
Mean (%)	0.0893	0.1208	0.2784	0.04727	0.5542	0.7293	0.04727	0.6067	0.04202	0.1956
	PPTg	PrL	PT	Re	Rh	RR	SG	SNC	SNL	Sub
Mean (%)	0.09754	0.1286	0.5098	0.136	0.06283	0.0841	0.1133	0.343	0.2705	0.01051
	SubB	TE	TU	VIS	VMH	VPM	VTA			
Mean (%)	0.5463	0.02364	0.005253	0.7244	0.2721	0.1167	0.07407			

Table S2. Details of statistical analysis.

Fig. 6A	Two-way ANOVA	Sidak's multiple comparisons test	*P*-value

	VGLUT2$^+$ vs. PV$^+$		< 0.0001
		AAA	adj. $P >$ 0.9999
		AI	adj. $P >$ 0.9999
		Au	adj. $P =$ 0.9105
		BST	adj. $P >$ 0.9999
		CA1	adj. $P <$ 0.0001
		CPu	adj. $P <$ 0.0001
		DR	adj. $P >$ 0.9999
		Ect	adj. $P >$ 0.9999
		GI	adj. $P >$ 0.9999
		IPAC	adj. $P >$ 0.9999
		LEnt	adj. $P =$

			0.1925
		MCPO	adj. $P >$ 0.9999
		MG	adj. $P >$ 0.9999
		MO	adj. $P >$ 0.9999
		Pir	adj. $P <$ 0.0001
		Po	adj. $P >$ 0.9999
		PRh	adj. $P =$ 0.8415
		PVT	adj. $P >$ 0.9999
		TeA	adj. $P >$ 0.9999
		SI	adj. $P =$ 0.9986

Fig. 6B	Two-way ANOVA	Sidak’s multiple comparisons test	P-value
	PKC-δ^{+} vs. SST^{+}		< 0.0001

		Acb	adj. $P =$ 0.9954
		AI	adj. $P >$ 0.9999
		Au	adj. $P =$ 0.1524
		BST	adj. $P >$ 0.9999
		CA1	adj. $P <$ 0.0001
		DI	adj. $P >$ 0.9999
		DM	adj. $P >$ 0.9999
		DpMe	adj. $P >$ 0.9999
		Ect	adj. $P >$ 0.9999
		IPAC	adj. $P >$ 0.9999
		LEnt	adj. $P =$ 0.9401

		LH	adj. *P* > 0.9999
		LPO	adj. *P* > 0.9999
		LS	adj. *P* > 0.9999
		MG	adj. *P* > 0.9999
		MPA	adj. *P* = 0.9989
		Pir	adj. *P* > 0.9999
		Po	adj. *P* > 0.9999
		PRh	adj. *P* > 0.9999
		PSTh	adj. *P* > 0.9999
		PVT	adj. *P* = 0.6319
		S	adj. *P* = 0.9995

		TeA	adj. *P* = 0.0023
		SI	adj. *P* > 0.9999

Fig. 6C	Two-way ANOVA	Sidak's multiple comparisons test	*P*-value
	VGLUT2$^+$ vs. PV$^+$		< 0.0001
		Isocortex	adj. *P* > 0.9999
		Pallidum	adj. *P* = 0.9873
		Striatum	adj. *P* < 0.0001
		Hippocampal formation	adj. *P* > 0.9999
		Thalamus	adj. *P* = 0.9997
		Hypothalamus	adj. *P* > 0.9999
		Midbrain	adj. *P* > 0.9999
		Olfactory areas	adj. *P* =

			0.0155

Fig. 6D	Two-way ANOVA	Sidak's multiple comparisons test	*P*-value
	PKC-δ^{+} vs. SST^{+}		< 0.0001
		Isocortex	adj. *P* = 0.0114
		Pallidum	adj. *P* > 0.9999
		Striatum	adj. *P* < 0.0001
		Hippocampal formation	adj. *P* = 0.0250
		Thalamus	adj. *P* = 0.8942
		Hypothalamus	adj. *P* = 0.3411
		Midbrain	adj. *P* = 0.9546
		Olfactory areas	adj. *P* > 0.9999

Table S3. Numbers of starter cells and input cells.

Mouse number	No. starter cells	No. input cells	Input/starter cells
VGLUT2-#1	71	2902	40.87
VGLUT2-#2	103	3325	32.28
VGLUT2-#3	53	2394	45.17
VGLUT2-#4	71	3237	45.59
PV-#1	23	1523	66.22
PV-#2	28	2602	92.93
PV-#3	70	3650	52.14
PV-#4	26	1550	59.62
PKC-δ-#1	75	5436	72.48
PKC-δ-#2	95	11026	116.06
PKC-δ-#3	24	2554	106.42
PKC-δ-#4	40	4777	119.43
SST-#1	85	6127	72.08
SST-#2	159	13501	84.91
SST-#3	116	9519	82.06
SST-#4	86	3235	37.62
